# Tickle me, I think I might be dreaming! Sensory attenuation, self-other distinction, and predictive processing in lucid dreams

**DOI:** 10.3389/fnhum.2014.00717

**Published:** 2014-09-17

**Authors:** Jennifer M. Windt, Dominic L. Harkness, Bigna Lenggenhager

**Affiliations:** ^1^Theoretical Philosophy Group, Department of Philosophy, Johannes Gutenberg-University of MainzMainz, Germany; ^2^Institute of Cognitive Science, University OsnabrückOsnabrück, Germany; ^3^Department of Neurology, University Hospital ZurichZurich, Switzerland; ^4^Institute of Physiology and Zurich Center for Integrative Human Physiology (ZIHP), University of ZurichZurich, Switzerland

**Keywords:** agency, self-other distinction, dreaming, lucidity, tickling, self-tickling, sensory attenuation, predictive processing

## Abstract

The contrast between self- and other-produced tickles, as a special case of sensory attenuation for self-produced actions, has long been a target of empirical research. While in standard wake states it is nearly impossible to tickle oneself, there are interesting exceptions. Notably, participants awakened from REM (rapid eye movement-) sleep dreams are able to tickle themselves. So far, however, the question of whether it is possible to tickle oneself and be tickled by another *in* the dream state has not been investigated empirically or addressed from a theoretical perspective. Here, we report the results of an explorative web-based study in which participants were asked to rate their sensations during self-tickling and being tickled during wakefulness, imagination, and lucid dreaming. Our results, though highly preliminary, indicate that in the special case of lucid control dreams, the difference between self-tickling and being tickled by another is obliterated, with both self- and other produced tickles receiving similar ratings as self-tickling during wakefulness. This leads us to the speculative conclusion that in lucid control dreams, sensory attenuation for self-produced tickles spreads to those produced by non-self dream characters. These preliminary results provide the backdrop for a more general theoretical and metatheoretical discussion of tickling in lucid dreams in a predictive processing framework. We argue that the primary value of our study lies not so much in our results, which are subject to important limitations, but rather in the fact that they enable a new theoretical perspective on the relationship between sensory attenuation, the self-other distinction and agency, as well as suggest new questions for future research. In particular, the example of tickling during lucid dreaming raises the question of whether sensory attenuation and the self-other distinction can be simulated largely independently of external sensory input.

“… from the fact that a child can hardly tickle itself, or in a much less degree than when tickled by another person, it seems the precise point to be touched must not be known…”

(Darwin, 1859)

## Introduction

Why is it almost impossible to tickle oneself, and so easy to be tickled by others? And what can tickling tell us about the sense of agency, ownership and the self-other distinction? At least since Darwin, it has been thought that the inability to self-tickle—especially to the point of inducing laughter—is linked to the unpredictability and uncontrollability of other- as opposed to self-tickling. The advent of tickling machines enabled researchers to identify and isolate the relevant factors in an experimentally controlled manner. In a seminal study, Weiskrantz et al. (1971) devised an apparatus that could be used for active (motor command plus proprioceptive feedback) or passive (proprioceptive feedback *without* motor commands) self-tickling as well for being tickled by another person. They found that active self-tickling was least effective, with passive self-tickling being intermediate between active self-tickling and being tickled by another. This result, which has been confirmed in a number of follow-up studies (e.g., Blakemore et al., 2000b), suggests that sensory feed-forward information, but also proprioceptive feedback from the tickling hand are crucial for sensory attenuation during self-tickling and for the self-other distinction. The general idea is that sensory attenuation, in which the sensory consequences of self-generated actions are dampened, underlies the ability to distinguish between self and others (Blakemore et al., 2000a; Frith et al., 2000). Because the sensory consequences of self-produced tickling match our predictions and thus are unsurprising, they also *feel* less ticklish than the more unexpected tickles produced by others. Indeed, on this view, the felt ticklishness of other-produced tickles alerts us to the fact that we have been tickled by another, and not by ourselves.

### Tickling in a predictive processing framework

The theoretical framework proposed by predictive processing accounts now offers a new perspective on Darwin's claim that sensory attenuation during self-tickling depends on the predictability of the stimulus. According to this framework (e.g., Clark, 2013b; Hohwy, 2013), the brain is essentially involved in hypothesis testing and prediction error minimization, with prediction errors resulting from a mismatch between predicted and actual sensory input. While prediction error minimization has been suggested to operate on many different levels of the cortical hierarchy and to underlie a wide range of cognitive processes, including perception, beliefs, learning and attention to illusions, hallucinations and delusions (Mumford, 1992; Hohwy, 2010), there are principally two different ways in which it can be achieved. First, incoming sensory inputs can be used to optimize internal predictions (or generative models) about the brain's next possible states, as in perception. Second, action, or active inference, ensues when the organism changes its sensory inputs in order to better match its predictions (Friston et al., 2011). What the brain abhors, on this account, is surprise: the amount of surprise, or more technically free energy (Friston and Kiebel, 2009), signals that the internal predictions were insufficiently accurate or outright false. Less surprise, on this view, indicates a better fit of the internal models.

This view offers a new way of making sense of sensory attenuation during self-tickling. On the classical model, a copy (the so-called efference copy) of motor commands is used to compare the predicted and actual sensory consequences of self-generated movement; when the discrepancy is minimal, sensory attenuation occurs (Blakemore et al., 1998a,b, 2003; Frith et al., 2000). By contrast, predictive processing accounts do away with the need for an efference copy, suggesting that in ambiguous situations, the attribution of agency can be resolved by attending away from the consequences of self-generated movements. On this view, “sensory attenuation is a necessary consequence of reducing the precision of sensory evidence during movement to allow the expression of proprioceptive predictions that incite movement” (Brown et al., 2013, p. 413). Attenuation, in other words, is the phenomenal mark of self- as opposed to other-generated action.

A recent study (van Doorn et al., 2014) contrasted these two accounts by investigating self-tickling and being tickled by another person in a highly surprising context—namely an experimentally induced self-other confusion involving the illusion of having swapped bodies with someone else (cf. Petkova and Ehrsson, 2008) and of experiencing another person's hand as one's own (cf. rubber hand illusion; Botvinick and Cohen, 1998). The background idea was that this would be a way of testing whether confrontation with a highly non-standard, surprising situation might undermine the precision with which the exact pattern of proprioceptive and tactile feedback during self-tickling could be predicted—thus enabling it to feel more like being tickled by someone else. Whereas this would fit the classical efference copy model, van Doorn and colleagues' findings suggest that this is not the case: “even as participants shift their first-person perspective to someone else's, or experience having a baseball bat as a hand, or an invisible hand, there is no change in the characteristic pattern of feeling less tickle sensation when producing the touch themselves, and more tickle sensation when the touch is produced by someone else” (van Doorn et al., 2014, p. 8). The authors conclude that because sensory attenuation during self-tickling remains robust even in these highly surprising conditions, active inference, rather than context, is crucial for sensory attenuation, thus favoring predictive processing over the classical efference copy model.

### The phenomenal-functional characteristics of dreaming

In the following, we argue that dreams are a unique contrast condition for investigating the relationship between agency and the self-other distinction not just in specific experimental setups in waking participants, but across the sleep-wake cycle. In particular, the example of dreaming can extend existing work on sensory attenuation and the self-other distinction within the framework of predictive processing.

First, while paradigms investigating full-body illusions such as the body-swap illusion aim to disturb the mechanisms underlying the self-other distinction in healthy, waking subjects, dreaming involves a more profound and naturally occurring breakdown of the distinction between self and non-self, or between internally and externally generated sensory information. In the dream state, what is in fact an internally generated world-model—the dream world—is not experienced as self-generated, but simply as real (Metzinger, 2003; Revonsuo, 2006), typically including the experience of interacting with mind-independent characters and objects. Dreams are, in other words, *immersive spatiotemporal hallucinations* (Windt, 2010, 2015): they involve the robust sense of presence in a world that is experienced as real; yet, at the same time, this experienced world is only weakly constrained by sensory inputs from the sleeping subject's actual environment and is largely the product of internal signal generation, and hence hallucinatory. Because of this profound confusion of internally and externally produced stimuli, dreaming has even been suggested to be a model of delusional and hallucinatory wake states, such as those arising in schizophrenia (see Hobson, 1999; Gottesmann, 2006; see Windt and Noreika, 2011, for critical discussion).

Second and relatedly, social imagery is abundant in dreams, with non-self (usually human) dream characters being described in over 95% of adults' dream reports and the average dream involving 2–4 non-self dream characters (Kahn et al., 2000; see Nielsen and Lara-Carrasco, 2007, for details and further references). These are typically experienced as being highly realistic and clearly distinct from the self. Social interactions are actually even more frequent in dream reports than in randomly timed waking reports (McNamara et al., 2005) and are often experienced as emotionally engaging (Kahn et al., 2002). In particular, non-self dream characters are often experienced as having a mind of their own, with dream reports frequently describing cases in which the dreamer engages in theory-of-mind attributions by ascribing emotions, beliefs and desires to other dream characters (McNamara et al., 2007). This suggests that dreaming involves not only a breakdown of the distinction between internally and externally generated sensory information, but also specific disturbances in self-other distinctions.

Third, while both dreaming and wakefulness are characterized, on the phenomenological level of description, by the experience of interacting with a world, the transition from wakefulness to dreaming is accompanied by important functional changes. During the dream state, conscious experience is comparatively shielded from and only weakly constrained by external stimuli. While external stimuli are occasionally incorporated in dreams, the pattern of incorporation is often indirect, resembling sensory illusions rather than veridical perception (as in a dream of hearing a siren that is triggered by the sound of one's alarm clock; cf. Nielsen et al., 1995; see Windt, 2015, for theoretical discussion). Moreover, REM-sleep paralysis, or the near-complete absence of muscle tone during REM sleep, prevents the outward enactment of internally experienced movements (for a discussion of important exceptions involving dream-enactment behavior, see Schenck, 2005; Nielsen et al., 2009; Leclair-Visonneau et al., 2010). This unique phenomenal-functional configuration, as will become clear below, is particularly interesting from a predictive processing perspective.

### Predictive processes in dreams

Recent attempts to accommodate REM-sleep dreaming in a predictive processing framework suggest that alterations in the monitoring and generation of sensory predictions might be crucial to dreaming. As noted above, these accounts owe some of their attraction to the ambitious claim that not just veridical perception, but also imagination, hallucinations and nocturnal dreams are the outcome of a process of hypothesis testing and prediction error minimization. In this framework, dreaming, due to the comparative attenuation of external stimulus processing, has been described as a state in which hypothesis testing and prediction error minimization can be rehearsed and optimized (Clark, 2013a,b; Hohwy, 2013; cf. Hobson and Friston, 2012, 2014).

For the same reason, however, dreaming also presents a challenge for predictive processing accounts. Recall that the key claim of these accounts is that internal predictions are tested against incoming sensory stimuli, resulting either in the optimization of the internal, generative models themselves (as in perception) or in changing the incoming stimuli to better fit the internal models (as in active inference). In dreams, however, both types of processes are disturbed: because dreams unfold largely independently of sensory input and motor output, the crucial ingredient for either model optimization or active inference is lacking. Yet, because dreaming nonetheless involves the vivid phenomenology of perceiving and interacting with a mind-independent world rather than with one of our own making, both processes must be simulated, as it were, largely offline. Indeed, it has been suggested that dream bizarreness might result from the fact that dreams are largely unconstrained by external stimuli and hence by prediction errors, leading to the loss of representational accuracy, for instance of visual dream imagery (cf. Fletcher and Frith, 2009; Hobson and Friston, 2012). This does not explain, however, why large portions of dream experience are not bizarre, but are experienced as highly realistic (including, as noted above, non-self dream characters). This in itself is a remarkable computational achievement, suggesting that in the special case of dreaming, the processes of prediction error minimization and hypothesis testing are simulated largely internally, but nonetheless in a fairly realistic manner. Dreams thus offer a unique opportunity for investigating the interplay between hypothesis testing and prediction error minimization on the one hand and the sensory stimuli they are tested against, in standard wake states, on the other hand, suggesting that this relationship changes dramatically over the sleep-wake cycle.

### Self tickling in dreams?

In sum, the presented literature suggests that a transient breakdown in the ability to discriminate, on the level of phenomenal experience, between self- and other-generated actions, mediated by disturbances in the sense of agency and the precision of sensory predictions, might be crucial to the unique phenomenology of dreaming. Here, we suggest that questions about the process of hypothesis testing and prediction error minimization in dreams can be sharpened by focusing on the special question of why, in dreams, self-produced actions are experienced as if they were caused by others. Again, sensory attenuation for self-tickling as opposed to being tickled by another is a promising example. In particular, schizophrenics, unlike healthy participants, are able to tickle themselves (Blakemore et al., 2000a,b), presumably due to a disturbance in self-other distinctions. Similarly, Blagrove et al. (2006) found that participants awakened from REM sleep dreams are able to tickle themselves, which they explained by saying that “a deficit in self-monitoring and a confusion between self- and external-stimulation accompany REM dream formation” (Blagrove et al., 2006, p. 291).

The logical next question to ask, we suggest, is whether it is possible to tickle oneself in dreams. Here, it is important to note that the evidence presented by Blagrove and colleagues is indirect at best, as the effect was only observed after awakening and not during the dream state itself. Moreover, participants were only asked about the presence or absence of dream recall, but the content of their dreams was not analyzed. This points to an important methodological limitation, namely the practical impossibility of obtaining systematic ticklishness ratings for self- as compared to other-administered tickles during dreams. Lucid dreams, however, are an important exception, as they involve not only insight into the fact that one is now dreaming, but often also the ability to control the dream narrative, including the actions of non-self dream characters (LaBerge, 1985, 1990; Voss et al., 2013). Lucid insight into the fact that one is dreaming often coexists alongside vivid visual and motor hallucinations and social imagery, sometimes even leading lucid dreamers to think they are sharing their dream with another (Levitan, 1994). This is important, because it suggests that the disturbances in self-other distinctions that characterize nonlucid dreams largely remain intact in lucid dreams.

Our study aimed to exploit this fact by asking participants to contrast self- and other-administered tickles in three conditions: wakefulness, imagination, and lucid dreaming. Based on theoretical considerations on lucid dreams, but also on findings on self-tickling in healthy participants, schizophrenics, and following REM-sleep dreams, we predicted that while our participants would rate other-administered tickles as more ticklish than self-administered ones in wakefulness (*prediction 1*), this difference would be diminished in dreams (*prediction 2*). We also expected that in dreams, self-tickling would feel more like being tickled by another than like self-tickling in wakefulness (*prediction 3*). By contrast, we expected the distinction between self- and other-administered tickles to be preserved for imagined tickles (*prediction 4*), though we expected that both would be rated as less ticklish than their actual (and dreamed) counterparts (*prediction 5*).

## An explorative study of self-tickling in lucid dreams

This explorative online study aimed to rate how ticklish it feels to tickle oneself as compared to being tickled by someone else in three different conditions: actual self-tickling vs. actually being tickled during wakefulness; imagined self-tickling vs. imagining being tickled; and self-tickling vs. being tickled by another in a lucid dream.

### Participants

Participants were recruited via a German Internet platform for lucid dreamers (www.klartraum.de). Sixty-one persons participated in the first part of the study (questionnaire on actual and imagined tickling in wakefulness), but only 9 participated in the second part (tickling in lucid dreams). From our data we cannot judge whether this high drop-out rate was due to the difficulty of the task or the time-consuming nature of the study as whole. We did, however, ask participants to fill out the lucid dreaming questionnaire even if they did not manage to tickle themselves in their dream. Out of the 9 dream responses, 7 (4 female, average age 20.7) were able to complete the task and were thus included in the analysis.

### Procedure

The experiment was entirely web-based. Written instructions were given to the participants before they started the experiment. Participants were instructed to complete the experiment in two sessions. Actual tickling and imagined tickling were performed in a first session during the daytime, followed by dream tickling in a second session during a lucid dream. In all conditions, participants were asked to use (or imagine using, respectively) a feather, brush or a similar tool to first tickle their own foot, then to ask (or imagine asking) someone else to tickle their foot. Immediately after each task (respectively after waking up from a lucid dream), they completed an online questionnaire, adapted from the study conducted by Blagrove et al. (2006), in which they were asked to rate how “intense,” “ticklish,” “pleasant,” and “irritating” the stimulation felt on a discrete scale from 0 (not at all) to 10 (extremely). For the dream condition, they were additionally asked to give a free dream report (see supplementary material). In order to minimize the risk of forgetting, we emphasized the importance of filling in the questionnaire and reporting their dream immediately after awakening.

### Results

**Figure 1:**
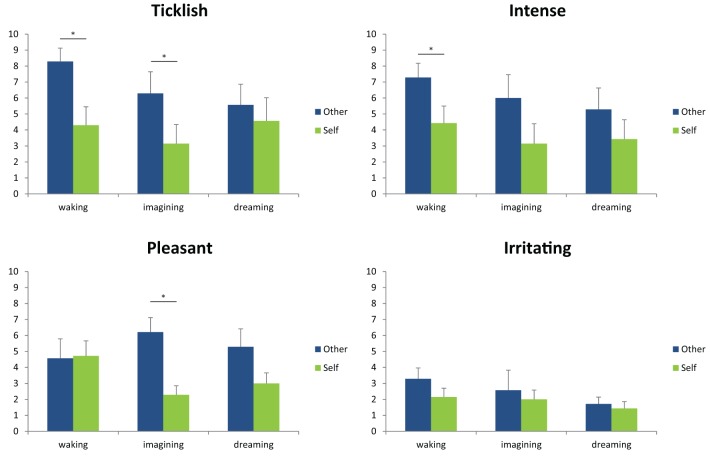
**Mean and standard error of the participants' ratings for each of the four scales in the three different conditions (waking, imagining, dreaming)**. ^*^Indicates *p* < 0.05 according to an uncorrected, non-parametric comparison between ratings for self-tickling and ratings for being tickled by another person.

The results are depicted in Figure 1, which shows mean and standard errors for each of the four scales in the three different conditions (waking, imagining, dreaming). Uncorrected wilcoxon tests (see Table 1) were done for each scale in each of the conditions in order to test whether there was a difference between self-tickling and being tickled by another person.

**Table 1:**
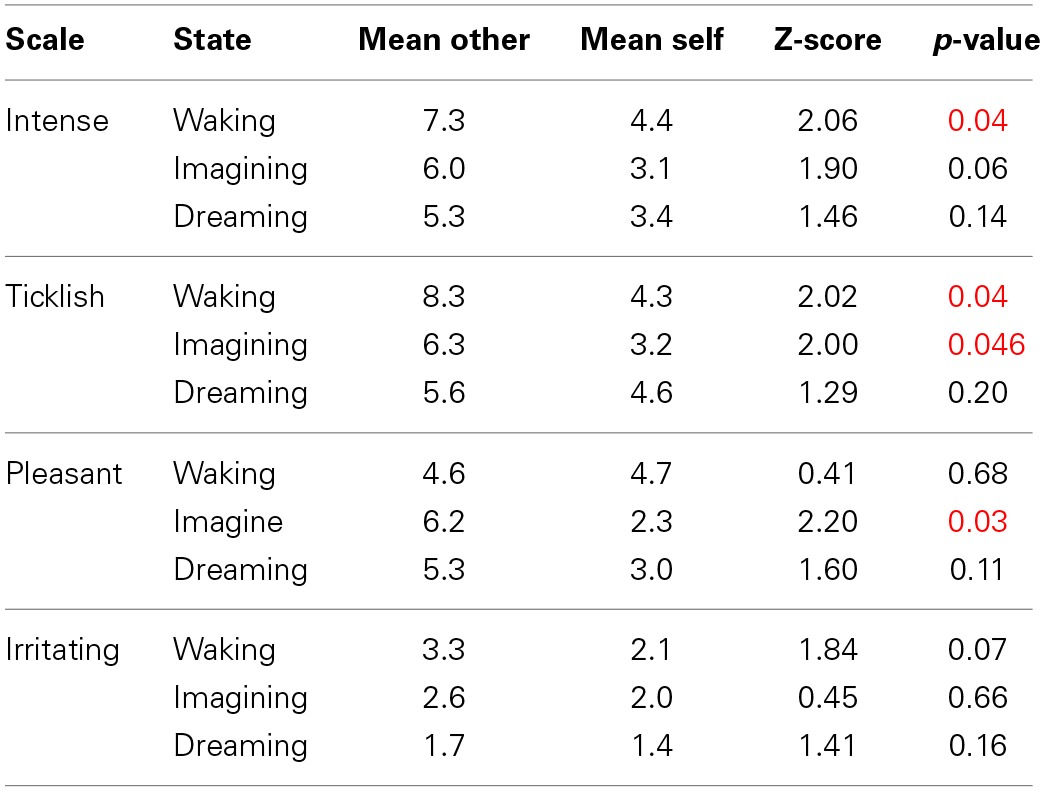
**Results of the Wilcoxon comparisons**.

*Red color = significant value (p < 0.05)*.

Confirming previous findings (e.g., Weiskrantz et al., 1971) and in line with *prediction 1*, participants' ratings of the ticklishness of other-administered tickles were higher than for self-tickling when the task was performed during wakefulness. A similar pattern was found for imagined self- and other-administered tickling, though both had a lesser absolute intensity than actual tickling (thus confirming *predictions 4* and *5*). This makes us confident that participants performed the test correctly and that the method was sufficient to replicate the results found by a number of existing studies. By contrast, during lucid dreams, and in line with *prediction 2*, we found no significant difference between self- and other-administered tickling. Interestingly, however, ticklish sensations in dreams still felt less ticklish than actually being tickled by another person during wakefulness and were comparable to waking self-tickling (Wilcoxon test, *Z* = 0.82, *p* = 0.41)—thus contradicting *prediction 3*. This effect was specific to ticklishness ratings, and dream tickles were rated as similarly intense, irritating and pleasant as imagined and/or actual tickles. Our highly preliminary conclusion is that both being tickled and tickling oneself, at least in a lucid dream, feel much like tickling oneself in wakefulness, but weaker than being tickled by another. This, in turn, suggests that in the special case of lucid control dreams, sensory attenuation characterizes not just self-administered tickles, but also those experienced as being administered by another. This stands in interesting contrast to the findings that schizophrenic participants rate self-tickling as being as intense as being tickled by another, and that the same is true for participants who have awakened from (presumably nonlucid) REM-sleep dreams.

## Limitations

Clearly, this study is subject to important limitations and the results should be taken with caution. Yet, we think that considering these in detail is interesting in itself, because it helps illustrate what we take to be the larger theoretical implications of this study. Though this may sound somewhat paradoxical, we think that the value of our study lies, in part, in the insights that can be derived from a careful consideration of what it did *not* show, and why. Indeed, this is also why we take the main value of this study to be of a theoretical rather than of an empirical nature. In particular, a discussion of these limitations also suggests a number of specific challenges and questions for future research.

### Practical and methodological limitations

To begin with, there are a number of practical and methodological limitations. Due to the demanding nature of the task, only a very small number of participants succeeded in completing the tickle-test in a lucid dream. Because this was an online study, we could not control whether the task was indeed carried out according to our instructions (though reports no. 4 and 5 suggest that this was the case), which sleep stage the lucid dreams occurred in, or how soon after awakening participants actually reported their dreams. This situation could be improved by conducting a laboratory study, insisting on signal verified lucid dreams and obtaining polysomnographic measurements to determine the sleep stages in which the dreams occurred (cf. LaBerge et al., 1981).

Furthermore, unlike the studies of self-tickling in waking participants, we were not able to use a tickling machine and thus to standardize the procedure. Rather, as shown by the dream reports, our participants dreamt up different tickling devices, such as wooden spoons, pens, or branches (cf. reports no. 1, 4, 6) and were also occasionally tickled elsewhere than on the foot (cf. reports no. 5, 8). A number of dream reports describe difficulties with dream-character compliance, such that dream characters refused to carry out the tickling task or poked rather than tickled the dream self (cf. report no. 1). Some dream reports are also too short to be sure whether dreamers were really lucid (cf. reports no. 3, 7, 8), and even when lucid, participants occasionally forgot to carry out the task (cf. report no. 9).

Expectation may have also biased our results. For instance, Giguère and LaBerge (1995) found that pinching in a lucid dream was not really painful, possibly due to expectation and motivation bias; moreover, at least one dream report (cf. report no. 2) suggests that the dreamer was theorizing about the outcome and implications of the experiment even during the lucid dream. Yet, the fact that ticklish-ratings for lucid dreams did not simply mirror ratings for actual and imagined tickling and specifically that the characteristic gap between self- and other administered tickles was preserved during imagined, but obliterated during dreamed task performance suggests that our study nonetheless tapped into a genuine difference.

### Theoretical limitations

A further limitation that is not specific to our study but characterizes lucid dream research in general is that the generalizability of results from lucid to nonlucid dreams is unclear. Indeed, it is possible that *prediction 3*, which was contradicted by our study, accurately characterizes nonlucid dreams. Because the phenomenal property of agency and the resulting ability to control both one's own and others' actions differ strongly between lucid and nonlucid dreams (Metzinger, 2003; Windt and Metzinger, 2007; Voss et al., 2013), and because of the suggested link between agency and sensory attenuation, it could well be that in nonlucid dreams, there would be no sensory attenuation for self-tickling.

A first step toward answering this question might be to compare ticklish sensations after waking up from lucid as compared to non-lucid dreams. If the attenuation of ticklish sensations in lucid dreams is indeed related to the increased sense of agency that characterizes lucid dream control, then one might expect both self- and other-administered tickles to be attenuated even after awakening from a lucid dream. Alternatively, the pattern observed in dreams might also be reversed, and participants awakened from a lucid dream might show the same ticklish ratings as participants awakened from nonlucid REM-sleep dreams, namely an increased ability to tickle themselves. It could also be the case, however, that after awakening from a lucid dream, ticklish ratings are the same as in standard wakefulness, but different from the pattern observed following nonlucid REM-sleep dreams. Indeed, lucid dreams are often described as involving a shift toward wake-like cognitive activity and agentive control and might even be regarded as subjective states in a much stronger sense than nonlucid dreams (Metzinger, 2003; Windt and Metzinger, 2007). It has also been suggested that lucidity occurs during a hybrid state between nonlucid REM-sleep dreams and wakefulness (Voss et al., 2009). Whatever the outcome, contrasting ticklishness ratings after awakening from lucid and nonlucid dreams might tell us something about the relationship between lucid insight, agency and sensory attenuation, as well as about the generalizability of our results from lucid to nonlucid dreams.

## Discussion

Given the limitations discussed above, the results of our study are highly preliminary. Yet, we think they give rise to a number of interesting, albeit speculative, considerations, as well as to some new hypotheses and perspectives for future research. In order to describe these in a maximally clear manner, we will assume, *purely for the sake of argument*, that our results had been substantiated by further studies. Skeptical readers are invited to regard the following as a theory-based thought experiment loosely inspired by some preliminary empirical observations.

### Does sensory attenuation really underlie the self-other distinction in dreams?

Even if they are taken at face value, it is important to note that the interpretation of our results is hampered by an underlying theoretical ambiguity. Spelling this out in some detail is instructive, because it helps illustrate a more general difficulty in comparing dreams and wakefulness. This is especially important given our claim that the example of lucid dreaming extends research on sensory attenuation in wakefulness. So far we have assumed that the weak ticklishness ratings found in our study are indeed an example of sensory attenuation specific to self-generated actions. However, because dream actions unfold largely independently both of the actual execution of dream movements (with the exception of dream-enactment) and of appropriate proprioceptive feedback, it is not clear that it makes sense to say that in dreams, the consequences of self-produced actions are attenuated in the first place. Moreover, while dreams typically involve the experience of phenomenal selfhood, or of being or having a self, bodily experiences are characteristically underrepresented in dreams, and body and body-part representations can also differ from the waking body (cf. report no. 4, which describes that the dreamer's toe looked like a banana, as well as difficulty controlling leg movements; for details and further references, see Windt, 2010, 2015). Consequently, it is possible that the attenuation of ticklish sensations observed in our study is an artifact of the more general phenomenal-functional characteristics of bodily experience in the dream state. On this view, sensory attenuation would only be present for the sensory consequences of actual movements and would not be applicable to the case of dreamed actions unfolding independently of their outward counterparts.

We do not, however, think that this alternative explanation, in itself, offers an entirely satisfying account of our findings. To begin with, studies of lucid dreaming suggest that dream movements continue to be associated with muscle twitches in the respective limbs (LaBerge et al., 1981; Fenwick et al., 1984) as well as with activation of the sensorimotor cortex (Erlacher and Schredl, 2008; Dresler et al., 2011). Moreover, while touch, thermal and pain sensations are only rarely described in dream reports (Hobson, 1988), both lucid and nonlucid dreams do at least occasionally include vivid tactile or even pain sensations (e.g., Voss et al., 2011). This was also the case in at least some of the dreams reported by our participants, who described either varying degrees of ticklishness or other sensations such as pain (cf. reports no. 1, 4, 5, 6). Also, a questionnaire-based study similar to our own found that dream caressing was rated as having equal intensity as actual (but not as imagined) caressing (Giguère and LaBerge, 1995). It at least seems possible, then, that our results can be compared to sensory attenuation of the type that is otherwise specific to the sensory consequences of self-generated actions in wakefulness.

A recent review of the factors underlying sensory attenuation further supports the claim that sensory attenuation is not wholly determined by motor predictions. As Hughes et al. (2013) suggest, the ability to predict or even control the timing of sensory events may also modulate sensory attenuation. As most existing studies have not controlled for these factors, it is unclear, according to the authors, that sensory attenuation, for instance during self-tickling, is driven by motor rather than temporal predictions or temporal control. They also tentatively suggest that temporal predictions may play a role in explaining schizophrenics' hallucinations and delusions of control. This leads us to speculate that a similar factor might be driving our results in lucid dreams.

A first conclusion, then, would be that lucid control dreams are the special case in which sensory attenuation spreads to actions initiated by “others,” at least in the sense in which non-self dream characters are experienced as distinct from the self, thus dampening other-generated tickles to a level comparable to self-generated ones. It is noteworthy that in dreams, this is not, however, associated with a complete obliteration of the experienced self-other distinction. By contrast, in wakefulness, *illusory* feelings of agency, or the experience of being able to control another's actions (e.g., *vicarious agency* Wegner et al., 2004) typically also result in an illusory feeling of ownership for these actions and in disturbed self-other distinctions (Tsakiris et al., 2006). For fully lucid dreams, the situation seems to be different: even though in such dreams, dreamers *know* that they are dreaming and are aware that non-self dream characters (including their actions) are ultimately creatures of their own making, they still continue to experience these as clearly distinct from themselves (see also our dream reports). Contrary to what one might expect based on studies of vicarious agency and full-body illusions in wakefulness, in dreams, controlling a body does not, it would seem, induce one to experience this body as one's own.

A fascinating question that we at present have no answer for is how to explain this difference. In order to be able even to gesture toward an explanation, one would have to know whether agency and/or sensory attenuation for dream tickles is prior to self-other distinction of the type involved, for instance, in experiencing another dream character as distinct from oneself (i.e., the dream self), whether the opposite is true, or whether these processes are independent. Whereas in wakefulness, ownership seems, at least occasionally, to follow on the heels of agency (such as in motor versions of the rubber-hand illusion; see Tsakiris et al., 2006), it is also possible that the purely phenomenological distinction between dream self and non-self dream characters taps into more basic and robust processes.

### Sensory attenuation and self-other distinctions in dreams from a predictive processing perspective

The problem of how to describe the relationship between sensory attenuation and self-other distinctions in dreams can be nicely sharpened by describing it from the perspective of predictive processing. Recall that predictive processing accounts suggest that in dreaming as in waking, we only have access to our generative models, but are never in direct perceptual contact with the world. Hence, the direct comparison between these states within a predictive processing framework seems permissible—with the exception, noted above, that in dreams, the predictions are not kept in check by the outer world, thus being able to “roam free.” Conscious experience in dreams, then, may be seen as isolating our prior convictions from the ability to test them against incoming sensory input. On this view, dreaming is even more strongly constrained by our prior convictions about the world because we lack the means to check and adjust them to sensory input during perception and active inference.

Moreover, recent attempts to account for self-consciousness in a predictive processing framework highlight the probabilistic nature of self-representation, including the representation of one's physical body (Limanowski and Blankenburg, 2013; Apps and Tsakiris, 2014). What is experienced as the self is, on this view, highly plastic and constrained not only by low-level influences, such as multisensory stimuli and even interoceptive cues (on the latter, see Seth et al., 2012; Aspell et al., 2013; van Elk et al., 2014), but also by high-level processes such as long-term beliefs. In particular, as Apps and Tsakiris (2014, p. 92) put it, “the free-energy account argues that information prior to an event will nuance predictions about the likely sensory input, and when sensory input is received, the prior information biases the probabilistic inferences that are made causes of an event.” Self-other distinctions in dreams, on this view, reflect sensory predictions operating under non-standard conditions of highly unstable and mostly internally generated sensory information and driven to a considerable extent by long-standing and shorter-term contextual beliefs.

What, then, are the priors driving the experience of self-tickling and being tickled by another in dreams? One of these, it would seem, is the conviction that we cannot fully control, or at least not directly and via acts of will, any bodily agent other than ourselves. Indeed, given that participants were asked to control the actions of dream characters they were already experiencing as distinct from the self, this might explain why the task investigated in our study was so difficult to complete in a lucid dream—and perhaps even the low response rate and the varying success of our participants. Perhaps, the type of control exerted over non-self dream characters in lucid dreams is sufficient to induce sensory attenuation for ticklish sensations, but not to obliterate the experience that other dream characters are distinct from oneself—and perhaps, the very nature of the task prevented our participants from developing this stronger form of control in the first place. This is also borne out by the fact that lucid dream control is often incomplete or has unintended results (Stumbrys et al., 2012). Yet, another interpretation is also possible. In particular, a strong conviction driving these effects in lucid dreams might be that to the extent that one is able to control an agent, this agent cannot be fully distinct from oneself. This would plausibly lead the sensory results of movements generated by these agents—such as tickling—to be experienced similarly to instances of tickling oneself. As Apps and Tsakiris (2014) note, the mere expectation or predictability of a self-stimulus might be sufficient to lead to sensory attenuation. As being tickled by another in a lucid control dream is predictable, this might account for the spread of sensory attenuation to tickles generated by non-self dream characters. This also fits in well with the finding that authorship beliefs about the causes of sensory changes in the environment may be one of the factors underlying sensory attenuation (Desantis et al., 2012).

But yet another and perhaps even more basic prior is needed to explain why the self-other distinction is not obliterated completely in lucid control dreams. This is that at any given moment, there should not only be a self, but also no more than a single self. Indeed, dreams exacerbate the computational problem of determining which one among a number of different body models is the unit of identification (Metzinger, 2013) and hence experienced as the self. Recall that dreams are not only rich with social imagery, but also that input from the physical body, typically a primary source of information for self-representation (Apps and Tsakiris, 2014), is only intermittently available. Yet, it is telling that even in lucid control dreams, where multiple (visual) body models are simultaneously active and under one's own control, only one of these is typically experienced as being the self, whereas the others are experienced as distinct from the self. This fits in well with the finding that in wakefulness, instances of bi-location and of identification with more than one body-model at the same time are rare and typically unstable (as in heautoscopy; see Blanke and Mohr, 2005; see also Furlanetto et al., 2013). Research is only beginning to investigate the feeling of *dis*owning one's own body in full-body illusions, and again, there is some indication that the experience of owning a different body comes at the price of disowning one's own (Guterstam and Ehrsson, 2012). Taken together with our evidence from lucid control dreams, this suggests that at its most basic, the self-other distinction is driven neither by agency nor by multisensory integration, but by the assumption that there is always exactly one unit of identification, the self. Dreams thus might be a good research model for investigating the simplest form of phenomenal selfhood (cf. Windt, 2010, 2015; Metzinger, 2013) as well as the most basic forms of modeling and understanding others (for a discussion of the applicability of predictive processing to social cognition, see Limanowski and Blankenburg, 2013).

In addition, note that in lucid dreams, there is also an interplay of long-standing and probably largely unconscious expectations of the type described above, and short-term, unconscious and conscious expectations about the specific situation encountered in the dream (for the effect of unconscious priming on sensory attenuation, see Gentsch and Schütz-Bosbach, 2011). Lucid dream control is a learnable skill (Stumbrys et al., 2012), and the complexity of the tickling task investigated in our study leads us to expect that our participants were likely experienced lucid dreamers, equipped with specific expectations about lucid dreams in general and non-self dream characters in particular. Indeed, as suggested by report no. 2, at least one participant was considering the theoretical implications of the dream experiment even while dreaming. At the very least, our participants, to the extent that they were indeed lucid, knew that they were dreaming and that they were controlling non-self dream characters that were not in fact real. They also may have had specific background beliefs about the autonomy of other dream characters, their own ability to control them, etc. Hence, it is quite possible that these lucid-dream-specific convictions colored our results as well. Indeed, dream report no. 4 describes that when the dreamer was unexpectedly tickled by another dream character, this felt more ticklish than willing the non-self dream character to perform the tickle-test. Expectations may have also been driving the dreamer's discovery, in the same dream report, that, following an initially weak tickling sensation, he or she had a Band-Aid on the foot—almost as if the process of dream imagery production were automatically explaining away the unexpected weakness of the sensation. Seen from a predictive processing perspective, it thus seems possible that the role of expectation in lucid dreams was not so much, as indicated above, a limitation as a factor contributing to sensory attenuation for self- and other-administered tickles.

While it seems difficult or even near-impossible, for practical reasons, to tease these different factors apart in future studies of lucid dreaming, the way forward, we suggest, might be to create an experimental setup that could be performed with waking participants, but that would nonetheless mimic the situation involved in lucid control dreams as closely as possible. We suggest that this might be a fruitful way of evaluating the different explanations briefly sketched above and thus of extending existing research on sensory attenuation during self-tickling.

### The way forward? toward a new experimental paradigm

The question, then, is whether a similar effect, involving sensory attenuation for other-administered tickling, whilst leaving the phenomenological distinction between self and non-self intact, might exist in standard wake states as well. To begin with, note that in a sense, our explorative study can be regarded as the mirror image of the study conducted by van Doorn et al. (2014). While they asked whether swapping bodies with another enables one to tickle oneself, our study investigated not only whether one can tickle oneself in a dream, but also, at least implicitly, whether one can tickle oneself by controlling, indirectly and via thought, the movements of a non-self dream character. The waking analog to this situation in lucid dreams would be to create a virtual reality (VR) setup in which participants can be tickled by avatars that are under their voluntary control for an extended period of time, but without simultaneously identifying with them or experiencing ownership for their bodies and bodily actions.

How might this be done? Standard VR setups and full-body and body-part illusions rely heavily on multisensory and sensorimotor coherence (for a review, see Bohil et al., 2011). Here, e.g., synchronous visuotactile stimulation leads participants to experience a virtual body (Lenggenhager et al., 2007) or body part (Botvinick and Cohen, 1998) as their own. The same is true for setups in which participants control an avatar by making real-body movements (Slater et al., 2010). In order to mimic the situation in lucid dreams, a first step would be to dissociate bodily imagery from real-body movement. Indeed, several studies have used brain-computer interfaces to enable participants to control avatars or robots via bodily imagery (i.e., merely imagined movement; cf. Pfurtscheller et al., 2006; Friedman et al., 2007a,b), thus approximating the type of thought control involved in lucid dreams. Here, the general finding, once more in keeping with newer accounts of self-other distinctions in a predictive processing framework (cf. Apps and Tsakiris, 2014), is that even these more abstract, imagistic forms of control lead participants to identify with the avatar. In order to mimic lucid control dreams, then, something more would be needed. In particular, VR would have to create a situation in which participants, perhaps thanks to sensorimotor coherence and bodily agency, first identified with one avatar, and then were given the ability to additionally control, perhaps via bodily imagery within the dream, the movements of another, such that the non-self avatar were now acting toward the self, e.g., by tickling its foot. We would now, as in a lucid dream, have two different avatars, driven by different kinds of control (e.g., bodily imagery vs. real-body movement and sensorimotor contingency), only one of which would be the target of ownership and identification. One could then investigate in more detail and in a more carefully controlled manner whether this would result, as in our study, in sensory attenuation for being tickled by the non-self avatar—and one could thereby make progress on isolating and experimentally manipulating the relevant factors underlying agency, ownership and the self-other distinction, as well as participants' prior expectations, both conscious and unconscious. A careful prediction would be that once participants had been induced to identify with one avatar, the unit of identification should remain stable even as they gain the ability to control another, which would continue to be experienced as distinct from the self. In particular, they should not, we submit, simultaneously identify with more than one avatar at the same time.

Even beyond the delicate matter of self-tickling, this type of experiment might have profound theoretical implications. In particular, it might help sharpen, both conceptually and experimentally, the distinction between different types of agency, ranging from agency for bodily movement under conditions of appropriate sensorimotor coherence, to bodily imagery in the absence of real-body enactment and sensorimotor coherence, to, perhaps, more abstract and conceptual forms of control, such as simply willing the avatar to tickle one's foot. It might also shed light on the degree of precision of temporal and motor predictions required for bringing about sensory attenuation for the actions of a non-self character (e.g., in a dream or an avatar in a virtual environment) that is under participants' indirect control (for an excellent review of factors underlying sensory attenuation, see Hughes et al., 2013). And finally, it might help identify (and tamper with) general, longer-term as well as context-specific, shorter-term expectations about the ability to control others in natural and virtual environments. At the same time, this type of experimental setup, though inspired by our findings in lucid dreams, might circumvent some of the methodological difficulties encountered by our study.

### Sensory attenuation reversed: toward a new theoretical perspective

More generally, if our results are taken at face value, they suggest a new perspective on the investigation of sensory attenuation. Much existing research has tried to create conditions in which the attenuation of self-generated actions is obliterated, raising them to the level of other-generated actions and events. We submit that this research strategy could be complemented by attempts to isolate the conditions under which other-generated actions are dampened to the level of self-generated ones—but apparently without thereby being experienced as one's own.

Studies investigating agency and self-other distinction during joint action (cf. Sebanz et al., 2006) indicate that sensory attenuation is indeed modulated by social interactions. Weiss et al. (2011) presented the first-ever evidence that sensory attenuation is not exclusively determined intra-individually, but also modulated by social interactions. Intriguingly, they found that sounds generated in an interactive context in which another person was acting on the participant's request were significantly attenuated, suggesting that “the other person may become an integral part of one's own internal sensorimotor loop that then specifies the relation between one's own transmitting action, the other's responsive action and sensory consequence” (Weiss et al., 2011, p. e22723). They also found that attenuation was strongest for self-produced sounds generated, interestingly, on request of another, possibly “due to a kind of contrastive enhancement of self-agency in the interactive action context” (Weiss et al., 2011, p. e22723). Yet, this is not to say that the difference between self- and other-generated actions is wholly obliterated in social interaction. Recently, it has been suggested that even in joint actions, such as in ensemble music performance, sensory attenuation helps distinguish one's own contributions to a shared goal from that of others (Loehr, 2013).

One way of explaining the results of our explorative study, consequently, might be to say that lucid dream control over the actions of non-self dream characters leads to sensory attenuation for other-administered tickles because this involves an incomplete simulation of joint action, where the non-self character is incompletely distinguished from the self. If this is correct, an intriguing possibility is that one way of investigating sensory attenuation during joint action may be to investigate cases in which no social interaction is actually taking place, but where social interactions are either simulated internally, as in lucid dreams, or technologically, as in the hypothetical VR experiment sketched above.

## Conclusions

To conclude, can you tickle yourself in a dream? At least for the special case of lucid control dreams, the answer seems to be no. And neither, apparently, can anyone else. Given the limitations of our explorative study, this result might be somewhat too weak to constitute a genuine test of whether one is now dreaming or awake, and thus to provide a palpable alternative to the better-known pinching test. Even though the tickle-test will likely not convince the determined skeptic, we still think, however, that the main value of this result is to suggest a new theoretical perspective on the problem of sensory attenuation for self- and other-generated actions, as well as new questions for future research. In investigating the factors contributing to sensory attenuation, future studies might focus not just on self-generated actions and events, but might also investigate the conditions under which sensory attenuation spreads to the sensory consequences of actions generated by others than the self. It might also focus on cases of simulated as opposed to actual social interaction and investigate in more detail how sensory attenuation and self-other distinctions change when they are simulated largely offline, as in dreams.

Finally, note that this also leads to an interesting metatheoretical observation. This is that aside from their specific results, lucid dream studies, even of the wholly exploratory nature presented here, may be theoretically valuable even when, as in our case, they are too speculative to warrant any strong conclusions in their own right. In particular, one reason for being interested in lucid dreams, if we are correct, is that the theoretical discussion of lucid dreaming is a kind of playground for dreaming up new and theoretically interesting experimental setups and suggesting new perspectives for future research, for instance on virtual reality, full-body illusions, sensory attenuation and the self-other distinction. If this is all we have achieved with this paper, we think it will have been well worth its while.

### Conflict of interest statement

The authors declare that the research was conducted in the absence of any commercial or financial relationships that could be construed as a potential conflict of interest.
