# Supplementary material for: Tickle me, I think I might be dreaming! Sensory attenuation, self-other distinction, and predictive processing in lucid dreams
Source: Front Hum Neurosci. 2014 Sep 17;8:717. doi: 10.3389/fnhum.2014.00717 (PMC4166313; doi:10.3389/fnhum.2014.00717)
Supplement: Supplementary file 1 [file DataSheet1.PDF]

Supplementary material for the paper:

Tickle me, I think I might be dreaming! Sensory attenuation, self-other distinction, and predictive processing in lucid dreams

Jennifer M. Windt, Dominic Harkness, Bigna Lenggenhager

1.

Fell asleep at 02:00: I fell asleep consciously. I was in a workroom with other people, where laboratory tables were set up in a row. A woman in military dress was sitting at the laboratory table and talked to me: - Our general always tickled us before the attack (She puts her left leg on the table). It was very important (she gave me the wooden spoon). Since if you attack… - One moment, I interrupt her, let me logically sum up: I'm in a chemical laboratory, a woman in military dress with the voice of a man is speaking to me and telling me that her general always tickled her before attacking. Do you know what that means? It's a dream! – (She nods affirmatively) – And that means… - You're supposed to tickle me! – (I turn the wooden spoon in my hand) No, that means you have to tickle me! I sit down in an office chair and put my right foot onto the laboratory table. – Aha, a right-hander, she looks down in a know-it-all*ish* manner – (ha-ha, how can she know this…) She takes the wooden spoon and slams the sharp end of the spoon into my foot, in the vicinity of the big toe. – Oww! – I screamed – Are you mad? You're supposed to tickle me, tickling and not stabbing! – Oh – a sadistic smile appears on her lips – I must've mixed things up. (She closes in on the foot again) – No, no, I don't trust you anymore (I take the spoon from her). I will look for someone else. Someone else? I think shortly. It's my dream. Why do I have to go anywhere? I turn to the woman and wave with the wooden spoon, like with a magic wand. The room dissolves. I'm lying barefooted on a beach at the sea, looking at the sunset and the woman isn't a grimly military-cannon anymore, but a congenial woman in a bikini. I get out the wooden spoon and give it to her. While doing it I have to laugh, since the Russian painted wooden spoon does not match the Haitian surroundings… and wake up. Well, laughing while lucid dreaming is a waste of energy! Early morning: Again I'm on Haiti, this time with Michael Jackson. He's playing a song on the ukulele and is also dancing to it. I'm too lazy for any kind of test. Since the sunset is just bombastic. Oh this lucid-dream sky… Heartbreaking! A woman filled with light tells me: - Give me your hand. Come with me, you've suffered enough on this earth. Suffered? I give her my left hand and hide the wooden spoon behind my back with my right. The glowing woman guides me over the earth to a river. – Leave all your thoughts here. There, where we are going, they will not be able to serve you anymore. – Yes, I'm coming! (I collect all my courage and pull out the wooden spoon). But before I'd like to ask you to tickle my foot. – You're giving me terms? – Well… I mean… it would be nice. She turns around and glides across the water's surface to the other shore. I've had enough! With the power of my thoughts I pull her back and put the wooden spoon into her hand. – Here's the spoon! Tickle! Please! But the woman becomes paler and dematerializes.

2.

After a false awakening from a lucid dream, I stood in the hallway of my parents' house. I already thought to myself that I'm still dreaming and wanted to conduct the new online experiment. I heard my parents talking in their bedroom, a shimmer of light came forth under the doorsill. To be sure that I wasn't disturbing them at anything I did a Nose-RT [reality test]:

positive. So I went in and found them lightly dressed on their bed. I said that I don't want to disturb them long. They should only tickle me on the bottom of my foot. I held both my feet towards them and they began to tickle, my father my left foot, my mother my right one. At first I didn't notice anything but then it did tickle me a little bit. About the same way as when I tickled myself during waking. I thought about how this stands in accord with the finger-counting experiments (I wasn't able to remember everything exactly, but in the dream my thoughts seemed to be quite logical): "The old experiments suggested that dreamer and other dream characters share one consciousness. Why can my dream-parents then tickle me? I'm not schizophrenic! As always the results of the experiments do not match my experiences!" A little upset about these online experiments, I still tickled myself quickly on my right foot, felt almost nothing, and left my parents' room. I ran out of the house onto the street. I wanted to visit my neighbor. When I noticed that I was running, I tried to walk normally, but not to fixate my eyes on anything. After a couple of steps the dream nonetheless collapsed. I'm not sure if a fake or real awakening followed.

3.

I laughed.

4.

… I notice for no particular reason that this is a dream, with the light feeling "Oh that's what I had forgotten" (so a classic MILD [mnemonic induction of lucid dreams], even though when falling back asleep after going to the toilet I had tried a WILD [wake-induced lucid dream]; I applied the MILD-suggestion the night before: "At the next opportunity, I will think about that I'm dreaming"). I decide to go back to deal with the Turkish adolescent that threatened me with the long switchblade. On my way I think of my LD [lucid dream] plan to just take in the surroundings: As during the entirety of my preceding nonlucid dream, I'm in a large, palatial department store with restaurants and food joints on different levels. Unfortunately I don't remember to take note of the details, instead I'm philosophizing that before, in the nonlucid dream, the surroundings appeared entirely realistic, whereas now I notice that everything I don't direct my attention at remains somewhat vague, or that this contrast is stronger than in WL [waking life]. I meet my brother, I'm pleased, but don't have any particular conversational topic. He asks me something related to medicine and I give him the correct information. Then he draws a comparison to his own field of activity (musician). During this conversation we climb over a low-hanging barrier tape, which however can't honestly be intended to serve as a barrier. Again and again I experience stability problems, for example my legs don't obey me correctly, or the scenery becomes more undefined. I work against it successfully by staying calm and letting my vision sway. As I'm thinking that (including the nonlucid dream) I've been dreaming for a very long time and could still do something useful, I remember the tickle experiment. As I face my companion again it's WILDling. Immediately he's engaged. We're in a section for large concert grand pianos. I sit on a stool, lean back and take off the sock on my right foot. My large toe looks exactly like a banana. I lean back and hold my naked foot towards WILDling. He tickles me with his fingertips, it feels kind of weak. Then he notices that there is a broad Band-Aid on the bottom of my foot. I ask him to rip it off, he's got problems with doing so, I grab it myself and remove it with a strong jerk (no pain). He tickles me again – without any noticeable increase in intensity. Then he wants to conduct tickle experiment himself. I keep it to myself that he is a dream character and tickle him with my fingertips first on the naked bottom of his right, and then of his left foot, during which he is laughing and gives up in every instance after a few seconds – obviously a clear "10" on intensity. On the grand piano next to me (that has been

degraded to a desk) I search for a suitable object. The most brush-like was a pen with a cap that had an end like a spatula. Meanwhile WILDing tickles my foot unexpectedly – here the intensity is significantly higher, about level 6 (in the questionnaire I formed the middle value from both intensities). The chosen instrument however shows only little tickle-effect. I wake up unprepared and I have serious problems remembering all of the events and their order correctly. I stand up, sit down in front of the PC, put down my X's and am pretty upset about the fact that I have to type a detailed dream report before having breakfast ;-)

5.

I was running away from a monster (alien), together with other people. We were just in a staircase when I thought of the OEL [online experiment lucidity]. I must've been lucid before, but didn't do a reality check. I then asked the person (I don't remember the gender, I'm guessing early 20's) next to me if he/she could tickle me. Tickled me under my arm (armpit). I felt it as 'ticklish' up to 80% compared to waking. After, I tickled myself while walking (same location). Felt it as less intense (about 20% of the intensity compared to wakefulness from a different person). I noticed that it does tickle me a little bit, something that only works with tricks during wakefulness (Cross arms, cross legs and then tickle the bottom of the foot). Then I woke myself up and wrote this dream report.

6.

I became lucid last night (from good Friday to Saturday, 11.04.09). Reason: Inconsistencies, forgot. After riding my Harley some time I decided, after it broke, to do the tickle-test. I went into a nice house (semi-detached house) on the left side of the street. I was excited about the tickle-test and met, as wished, a beautiful woman in the house (estimated age about 19). I broke off a branch from a bush before the door, gave it to her, asked her to tickle me with it and I raised my left bare sole towards her. She agreed, smiled and swept the branch across my foot. Although I did feel the branch and the movement, it didn't tickle much. I didn't have to laugh or anything like that. I also tried tickling myself, ended up with an even more sobering effect. I thanked her, she hugged me and I woke myself up. Emotions: Anticipation, affection towards the woman, other than that none. Note: I already conducted the tickle test a couple of days ago, but I forgot the brush/feather and also to tickle myself. I was tickled by a boy beneath my arms, the feeling was extremely ticklish, ecstatic and I woke up […]

7.

I was lying in bed, chained, with lots of feathers and other things lying on a box. Across the bed was a large black closet . A man tickled me!

He tickled so hard that I had to laugh loud as the bottom of my feet were being tickled… terrible

8.

It was nice because a friend (f.) did it to get me to laugh… I was sad… and he cheered me up and poked me in the side… I had to laugh because it tickled..

Then he started to tickle my sides and my stomach and my feet… XD

9.

I had different dream sequences. Among other things I was at a wedding (watched a film before about that topic), in a bar with dark wooden furniture, there was music, everyone was happy and talked, laughed and danced, there was a delicious wedding cake with white buttercream. I was also in a good mood and happy.

Also I was taking the train (a dream from work, I work for a train company), witnessed the everyday life, so checked tickets, answered questions etc. The sun was shining outside.

And I was at a horse show (I have a horse). It was summer, and warm, the grass already bleached out a little. The atmosphere was very soothing, I sat on a bench and was delighted by all the beautiful and shimmering horses.

I was constantly thinking: oh great, finally you're dreaming, now you have to do the tickle-test. Then I looked around to find a person to help me and then every time something happened (an exciting jump, a passenger had a question etc.) and the thought was gone again. But I did have, when I imagined doing the tickle-test, the clear feeling on my right foot of how it felt to tickle myself with the feather from the previous test.
